# Supplementary material for: Inulin prebiotic reinforces host cancer immunosurveillance via ɣδ T cell activation
Source: Front Immunol. 2023 Feb 17;14:1104224. doi: 10.3389/fimmu.2023.1104224 (PMC9981629; doi:10.3389/fimmu.2023.1104224)
Supplement: Supplementary file 1 [file DataSheet_1.pdf]

Supplementary Data and Tables

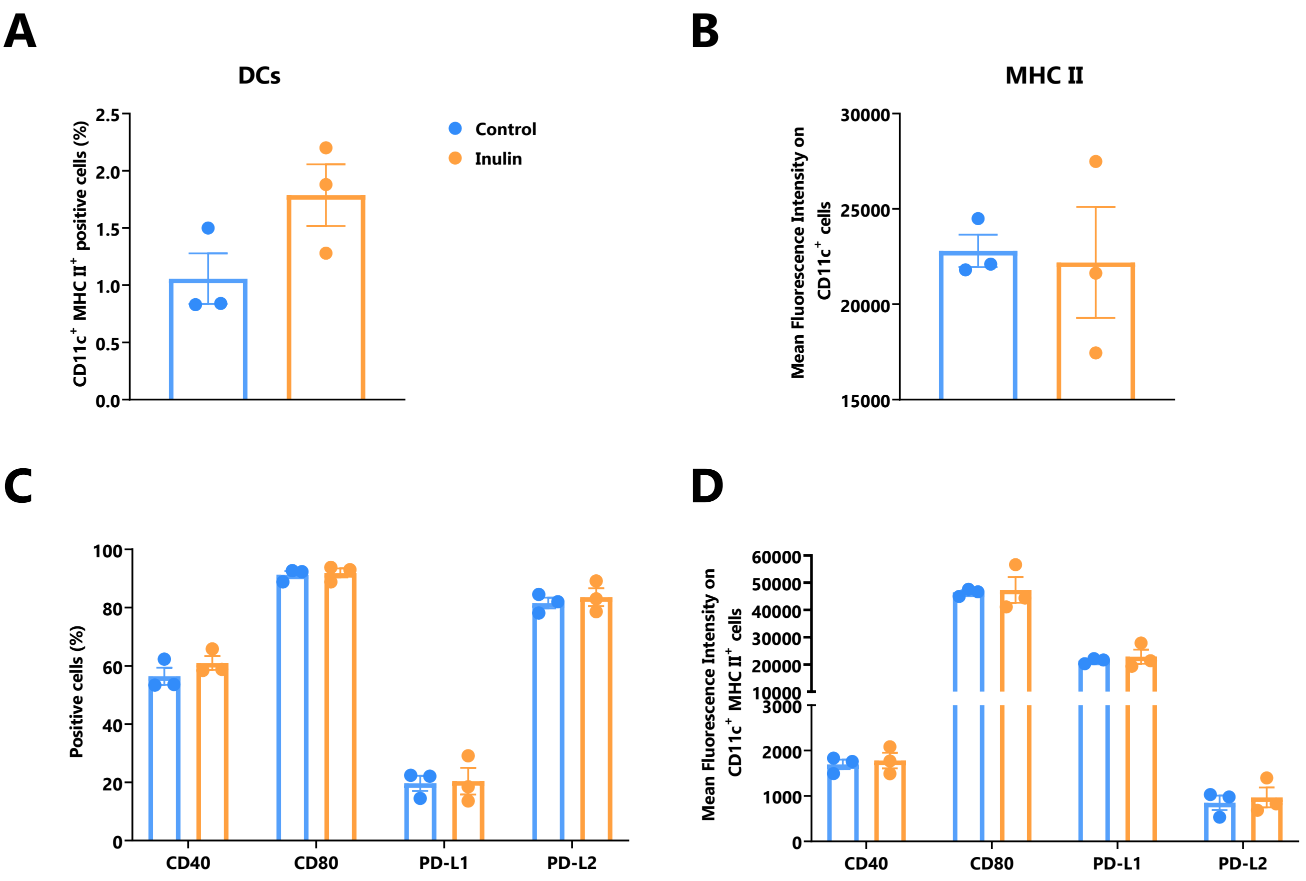

Supplemental Figure S1: Inulin-enriched regimen leads to higher DC infiltration in B16 OVA tumors.

(A) Frequency of Dendritic Cells (DCs, as defined as CD11c+MHCII+) among CD45^{+} cells in the B16 OVA tumor of mice treated as described in Figure 1A. (C) Frequency and (B-D) MFI of DC activation markers on DCs in the B16 OVA tumor of mice treated as described in Figure 1A. Graphs show the mean ± SEM.

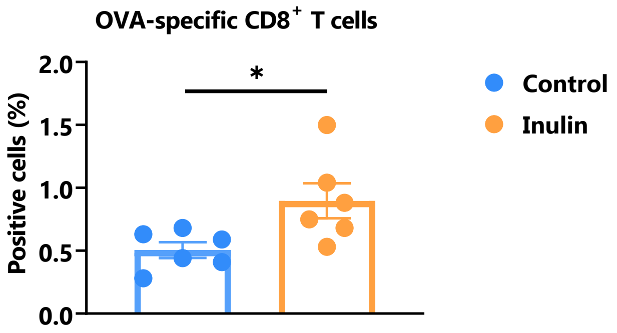

Supplemental Figure S2: Inulin potentiates antigen-specific CD8$^+$ T cell response after OVA PolyI:C immunization.

C57BL/6 mice were fed with a control diet or inulin-enriched diet (7.2% in drinking water) (n=12 mice per group) starting 15 days before s.c. immunization against OVA protein (500µg/mouse) adjuvantized with PolyI:C (50µg/mouse). Frequency of OVA-specific CD8+ T cells (defined as Dextramer+ among CD45+CD3+CD8+CD44+) in vaccine draining (inguinal) lymph nodes was analyzed 7 days post-immunization. Graph shows the mean ± SEM. *p < 0.05, by Mann-Whitney test.

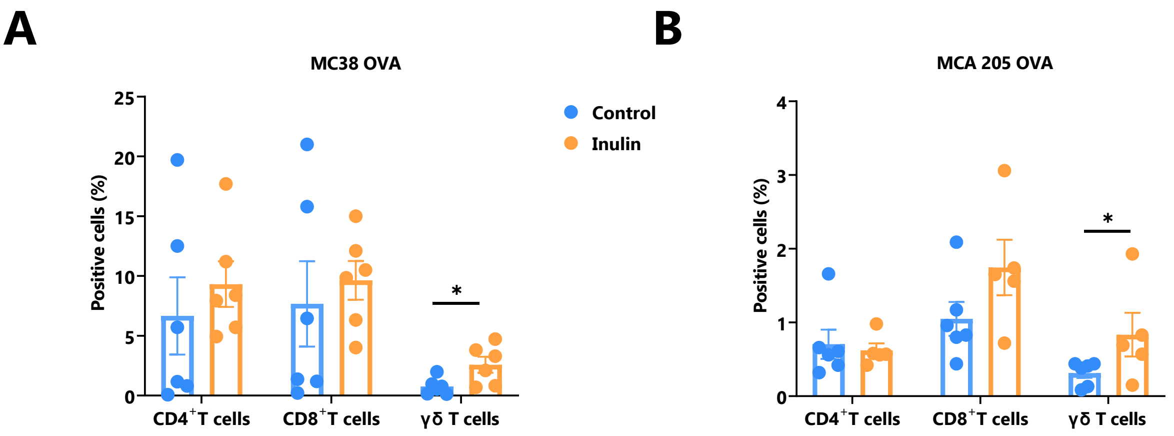

Supplemental Figure S3: Inulin regimen promotes γδ T cell infiltration in MC38 OVA and MCA 205 OVA tumor models.

C57BL/6 mice were fed with a control or an inulin-enriched diet (7.2% in drinking water) (n=6 mice per group) starting 15 days before subcutaneous (s.c.) inoculation of $5x10^5$ MC38 OVA colorectal cancer cells, or $2x10^5$ MCA 205 OVA fibrosarcoma cells (n=6 mice per group). Frequency of (A) MC38 OVA or (B) MCA 205 OVA tumor-infiltrated IFNγ-producing T lymphocytes. Graphs show the mean ± SEM. Statistically significant results are indicated by: *p < 0.05 by Mann-Whitney tests.

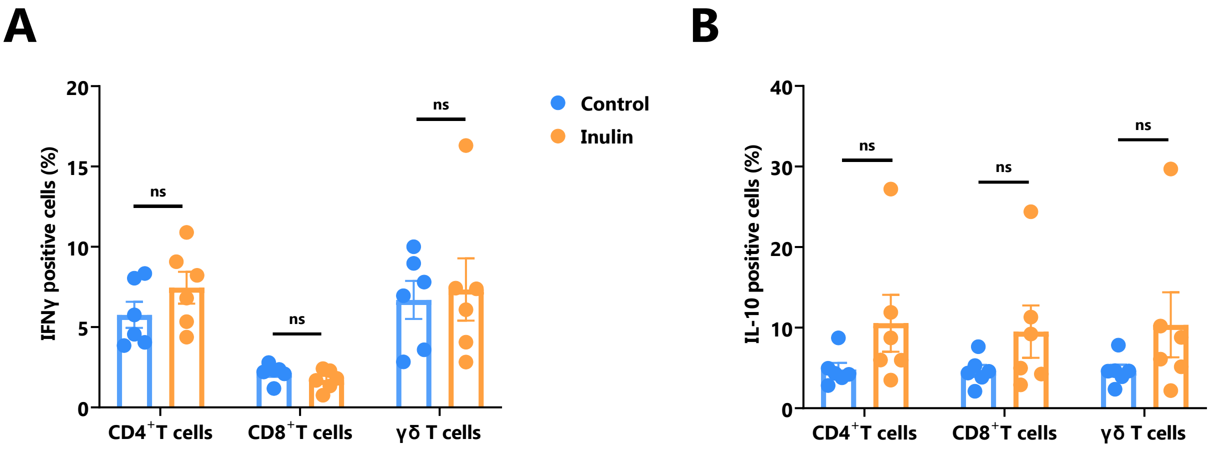

Supplemental Figure S4: Inulin consumption tends to increase IL-10 production by LPC in the colon.

C57BL/6 mice were fed with a control diet or inulin-enriched diet (7.2% in drinking water) (n=12 mice per group) for 15 days before the analysis of their gut immunity. Frequency of (A) IFNγ-producing or (B) IL-10-producing Lamina Propria T Cells (LPCs). Graphs show the mean ± SEM. ns = not-significant, *p < 0.05 by Mann-Whitney tests.

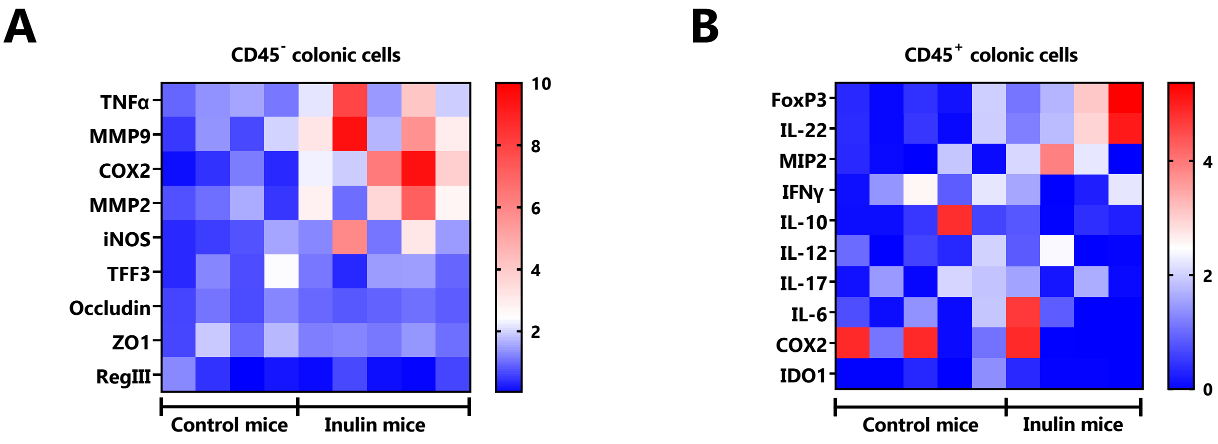

Supplemental Figure S5: Inulin diet impacts immune and epithelial cell inflammation in the colon.

qRT-PCR analysis of inflammation, tissue repair and tight junction -related genes in (A) $CD45^-$ and (B) $CD45^+$ colon cells sorted as described in (3E). Graphs show the expression levels of all the analysed genes.

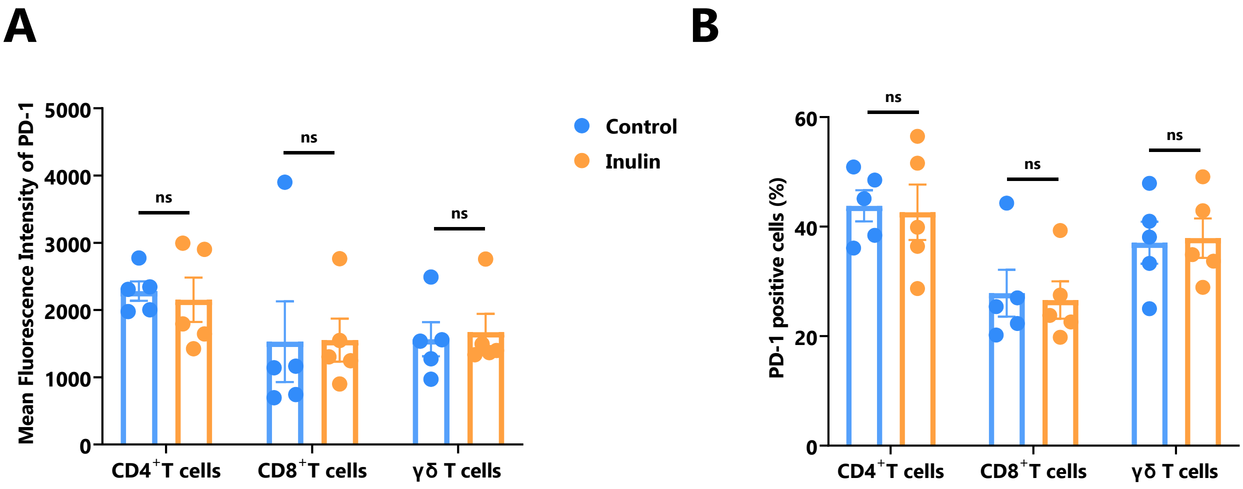

Supplemental Figure S6: Inulin does not affect PD1 expression by TILs in the B16 OVA tumor.
(A) Mean Fluorescence Intensity (MFI) of PD1 and (B) frequency of $PD1^{+}$ cells among $CD4^{+}$, $CD8^{+}$ and γδ T cells in the B16 OVA tumor of mice treated as described in Figure 1A. Graphs show the mean ± SEM. ns = not-significant by Mann-Whitney tests.

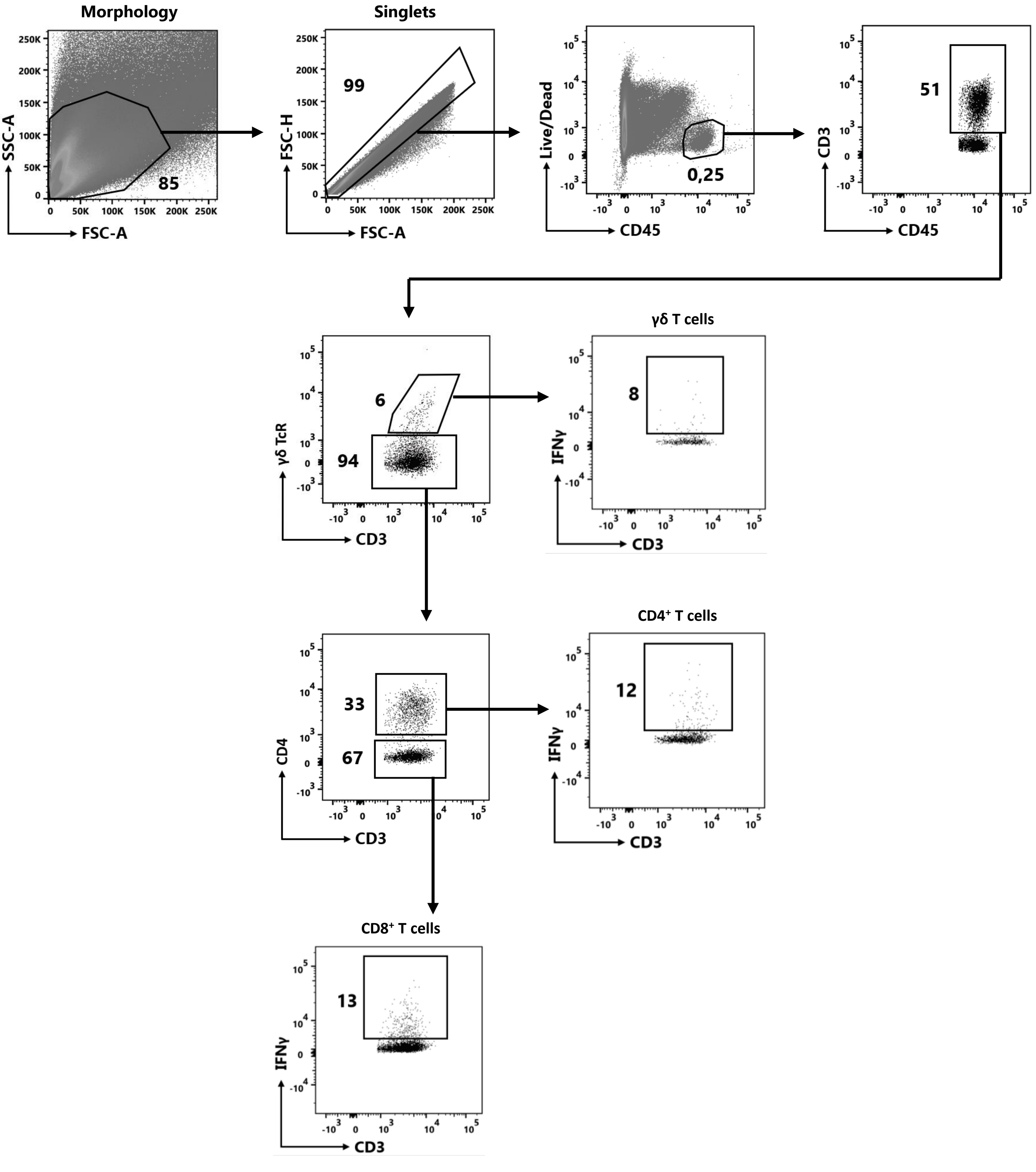

Supplemental Figure S7: Gating strategy.

Illustrative dot plots showing the gating strategy allowing the analysis of intracellular IFNγ production by TILs. The same gating strategy is used for IELs analysis.

Percentages (%)	Control	Inulin	Global
**Firmicutes**	59,61	57,11	58,30
**Bacteroidota**	37,87	35,87	36,83
**Desulfobacterota**	1,52	1,11	1,31
**Proteobacteria**	0,97	1,23	1,11
**Actinobacteriota**	0,03	4,67	2,46

Supplemental Table S1: Inulin diet alters microbiota composition Relative abundance of bacterial phyla in %. Means of 10-11 mice / group

Antibody	Source	Identifier
APC/Cy7 anti-mouse CD45	BD Bioscience	Cat# 557659, clone 30-F11, RRID:AB_396774
BV421 anti-mouse PD-1	BD Bioscience	Cat# 748268, Clone RMP1-30, RRID:AB_2872696
BV421 anti-mouse IL-17A	BD Bioscience	Cat# 563354, Clone TC11-18H10, RRID:AB_2687547
FITC anti-mouse CD3	Biolegend	Cat# 100204, Clone 17A2, RRID:AB_312661
PerCP/Cy5.5 anti-mouse CD4	Biolegend	Cat# 100434, Clone GK1.5, RRID:AB_893324
PE anti-mouse CCR9	Biolegend	Cat# 129708, Clone 9B1, RRID:AB_2073249
FITC anti-mouse CD11c	Biolegend	Cat# 117306, Clone N418, RRID:AB_313775
PerCP/Cy5.5 anti-mouse CD8	Biolegend	Cat# 100734, Clone 53-6.7, RRID:AB_2075238
BV421 anti-mouse PD-L2	Biolegend	Cat# 329616, Clone 24F.10C12, RRID:AB_2716087
APC anti-mouse PD-L1	Biolegend	Cat# 124312, Clone 10F.9G2, RRID:AB_10612741
PerCP/Cy5.5 anti-mouse MHC Class II	Biolegend	Cat# 107626, Clone M5/114.15.2, RRID:AB_2191071
PE/Cy7 anti-mouse CD80	Biolegend	Cat# 104734, Clone 16-10A1, RRID:AB_2563113
PE anti-mouse CD40	Biolegend	Cat# 124610, Clone 3/23, RRID:AB_1134075
APC anti-mouse IFNɣ	Biolegend	Cat# 505810, Clone XMG1.2 RRID:AB_315404
BV421 anti-mouse CD44	Biolegend	Cat# 103040, Clone IM7, RRID:AB_2616903
PE/Cy7 anti-mouse ɣδ TcR	eBioscience	Cat# 25-5711-82, Clone eBioGL3 (GL-3, GL-3), RRID:AB_2573464
PE anti-mouse IL-10	Thermo Fisher Scientific	Cat# 12-7101-82, Clone JES5-16E3, RRID:AB_466176

Supplemental Table S2: Antibodies used in flow cytometry.

Primer	Sequence (5' → 3')
GAPDH FW<br>GAPDH RV	GGTGAAGGTCGGTGTGAACG<br>CTCGCTCCTGGAAGATGGTG
β2M FW<br>β2M RV	GTATACTCACGCCACCCACC<br>TCCCGTTCTTCAGCATTTGG
IL-1β FW<br>IL-1β RV	TGATGAGAATGACCTCTTCT<br>CTTCTTCAAAGATGAAGGAAA
IL-6 FW<br>IL-6 RV	TAGTCCTTCCTACCCCAATTTCC<br>TTGGTCCTTAGCCACTCCTTCC
IL-10 FW<br>IL-10 RV	TCCTTAATGCAGGACTTTAAGGG<br>GGTCTTGGAGCTTATTAAAAT
IL-12 FW<br>IL-12 RV	CCTGGGTGAGCCGACAGAAGC<br>CCACTCCTGGAACCTAAGCAC
IL-17 FW<br>IL-17 RV	GCTCCAGAAGGCCCTCAGACTACC<br>CTTCCCTCCGCATTGACACAGC
TNF-α FW<br>TNF-α RV	AACTAGTGGTGCCAGCCGAT<br>CTTCACAGAGCAATGACTCC
IFNɣ FW<br>IFNɣ RV	GAACTGGCAAAAGGATGGTGA<br>TGTGGGTTGTTGACCTCAAAC
MIP-2 FW<br>MIP-2 RV	TCAATGCCTGAAGACCCTGC<br>CGTCACACTCAAGCTCTGGA

Primer	Sequence (5' → 3')
MMP-9 FW<br>MMP-9 RV	TGGGGGCAACTCGGC<br>GGAATGATCTAAGCCCAG
iNOS FW<br>iNOS RV	GTTGAAGACTGAGACTCTGG<br>ACTAGGCTACTCCGTGGA
Cox-2 FW<br>Cox-2 RV	GGGTTGCTGGGGGAAGAAATG<br>GGTGGCTGTTTTGGTAGGCTG
TFF-3 FW<br>TFF-3 RV	CCTGGTTGCTGGGTCCTCTG<br>GCCACGGTTGTTACACTGCTC
Occludin FW<br>Occludin RV	ACGGACCCTGACCACTATGA<br>TCAGCAGCAGCCATGTACTC
ZO-1 FW<br>ZO-1 RV	GGGGCCTACACTGATCAAGA<br>TGGAGATGAGGCTTCTGCTT
FoxP3 FW<br>FoxP3 RV	CCTATGGCTCCTTCCTTGGC<br>CCTTGGGTGCAGTCTTCCAG
RegIIIɣ FW<br>RegIIIɣ RV	TGGAGGTGGATGGGAATGGA<br>GCCACAGAAAGCACGGTCTA
IL-22 FW<br>IL-22 RV	GTGCTCAACTTCACCCTGGA<br>GGCTGGAACCTGTCTGACTG
IDO1 FW<br>IDO1 RV	TGGGACATTCCTTCAGTGGC<br>TCTCGAAGCTGCCCGTTCT

Supplemental Table S3: Sequence-specific primers used in qRT-PCR. FW = Forward sequence; RV = Reverse sequence.
